# Efficacy and safety of trastuzumab deruxtecan in the treatment of HER2-low/positive advanced breast cancer: a single-arm meta-analysis

**DOI:** 10.3389/fphar.2023.1183514

**Published:** 2023-06-22

**Authors:** Zongyu Li, Shangwen Guo, Haoyi Xue, Luying Li, Yuyuan Guo, Sinuo Duan, He Zhu

**Affiliations:** ^1^ Clinical Medical Research Institute, Zhanjiang Central Hospital, Guangdong Medical University, Zhanjiang, China; ^2^ School of Medicine, Shihezi University of China, Shihezi Xinjiang Production and Construction Corps, Shihezi, China

**Keywords:** trastuzumab deruxtecan, HER2-low/positive, advanced breast cancer, efficacy, safety

## Abstract

**Background:** Clinical trials have shown that the use of trastuzumab deruxtecan (DS-8201) alone is expected to provide novel therapeutic options for HER2-low/positive patients. Nevertheless, there are some variations in the efficacy of trial results, with potential risks at the safety level. Most DS-8201 trials in HER2 advanced breast cancer (ABC) have been conducted in the form of small-sample nonrandomized controlled studies, resulting in a lack of validated indicators to evaluate the efficacy and safety of DS-8201. Thus, this meta-analysis aimed to pool the results of various trials of DS-8201 alone to explore the efficacy and safety of DS-8201 in patients with HER2-low/positive advanced breast cancer.

**Methods:** Relevant studies were searched in seven databases, including Embase, PubMed, Web of Science, Cochrane Library, CNKI, VIP database and WanFang data, to collect single-arm studies on DS-8201 for HER2-low/positive ABC. MINORS was adopted for quality assessment and STATA 16.0 for data analysis.

**Results:** Ten studies involving 1,108 patients were included in this meta-analysis. As for the tumor response rate, the pooled ORR and DCR of all studies reached 57% (95% CI: 47%–67%) and 92% (95% CI: 89%–96%) respectively, and the pooled ORRs of the HER2-low expression group and the HER2-positive expression group were 46% (95% CI: 35%–56%) and 64% (95% CI: 54%–74%). Only the low expression group achieved median survival time, with a pooled median PFS and median OS of 9.24 (95% CI: 7.54–10.94) months and 23.87 (95% CI: 21.56–26.17) months, respectively. The most common treatment-related adverse events from DS-8201 were nausea (all grades: 62%; ≥ grade III: 5%), fatigue (all grade: 44%; ≥ grade III: 6%), and alopecia (all grades: 38%; ≥ grade III: 0.5%). Drug-related interstitial lung disease or pneumonitis occurred in 13% of the 1,108 patients, with only a 1% incidence of AE ≥ grade III.

**Conclusion:** The present study suggests that DS-8201 is effective and safe in the treatment of ABC with low or positive HER2 expression, providing additional relevant information for its clinical application. However, further strengthening of the pairs is needed, as well as more clinical studies to support individualized treatment.

**Systematic Review Registration:** https://www.crd.york.ac.uk/PROSPERO/, identifier CRD42023390316.

## 1 Introduction

Globally, breast cancer is the most prevalent malignancy among women, with approximately 2.26 million newly-diagnosed breast cancer cases and up to 680,000 deaths reported in 2020 (Sung et al., 2021). As the National Cancer Centre of China reported in January 2019, the number of new breast cancer cases in China increased by 24,100 from 2014 to 2015 (Chen et al., 2018; Zheng et al., 2019). Human epidermal growth factor receptor 2 (HER2) is a transmembrane protein encoded by the oncogene ErbB2, which causes the division and proliferation of cancer cells and is an immunohistochemical indicator in the pathology of breast cancer. The most commonly used criteria for HER2 detection in breast cancer tissues are *in situ* hybridization (ISH) assessment and immunohistochemistry (IHC) assessment, which can be classified as HER2 negative (HER2-0), HER2 low expression (IHC 1+ or IHC 2+ and ISH-) and HER2 positive (IHC 3+ or IHC 2+ and ISH+) (Wolff et al., 2018). A multicenter observational study based in Chinese hospitals showed that 21.6% of breast cancer patients were advanced at the time of diagnosis (Zeng et al., 2021). Currently, advanced breast cancer can be treated selectively with chemotherapy, endocrine therapy and anti-HER2 therapy depending on the patient’s health status, HER2 and hormone receptor (HR) status, etc. For triple-negative breast cancer among HER2-negative breast cancers, there are currently no immune drugs approved in China, and chemotherapy remains the main treatment, with options including anthracyclines and taxane. However, endocrine therapy with aromatase inhibitors combined with CDK4/6 inhibitors may be preferred for patients with advanced postmenopausal HR-positive/HER2-negative breast cancer (National Health Commission Of The People’s Republic Of China, 2022). For HER2-positive and low-expression breast cancers, targeted anti-HER2 therapy may be the cornerstone of comprehensive treatment. Anti-HER2 therapy is essential for HER2-positive advanced breast cancer, with 20%–30% of all breast cancer patients diagnosed as HER2-positive (Waks and Winer, 2019). The standard first-line treatment regimen is dual-targeted therapy (trastuzumab and pertuzumab) in combination with taxanes (National Health Commission Of The People’s Republic Of China, 2022; Giordano et al., 2022; Cardoso et al., 2014), but it is not curative for locally advanced or metastatic disease, with most patients experiencing disease progression (Harbeck et al., 2019). The EMILIA phase III clinical study of trastuzumab emtansine (TDM-1) solidified TDM-1 as the international standard of care for second-line anti-HER2 therapy (Verma et al., 2012). However, there is no unified third-line regimen after the failure of TDM-1 treatment, and all the available options have limited benefits and disappointing prognosis, leaving the next stage of therapy in a dilemma. Meanwhile, almost half (45%–55%) of diagnosed breast cancer patients have low HER2 expression (Tarantino et al., 2020), whereas anti-HER2 therapeutic agents including trastuzumab, lapatinib, and TDM-1 have failed to benefit patients with low HER2 expression due to a lack of potent targets (Press et al., 2008; Burris et al., 2011; Fehrenbacher et al., 2020). Therefore, breakthrough drugs are urgently needed for weak targets of HER2 low expression ABC and for disease progression after HER2-positive ABC treatment.

In late 2019, trastuzumab deruxtecan (DS-8201) was approved for the first time as a new targeted agent for the treatment of breast cancer by the U.S. Food and Drug Administration (FDA). DS-8201 is a novel HER2-targeted antibody-coupled drug that consists of a humanized HER2 monoclonal antibody (MAAL-9001). It causes a “bystander killing effect” by releasing a unique potent drug-carrying dexatecan derivative. Dexatecan derivative is a potent inhibitor of DNA topoisomerase I and can be used as a payload for antibody-drug conjugates (ADCs) to target HER2 that penetrates surrounding cells, leading to the apoptosis of the target and neighboring tumor cells (Nakada et al., 2016; Ogitani et al., 2016; Nakada et al., 2019). It is heartening to note that on 24 February this year, the Chinese National Pharmaceutical Administration (NMPA) announced the latest approval of Trastuzumab deruxtecan (DS-8201) in China for the treatment of unresectable or metastatic HER2-positive adult breast cancer patients who have received one or more anti-HER2 drugs in the past. In the DESTINY-Breast03 trial, the DS-8201 group achieved a disease control rate (DCR) of 97% in patients with HER2-positive advanced breast cancer, and an objective response rate (ORR) of 79.7% (95% CI: 74.3%–84.4%) of tumors, more than double that of the TDM-1 group (Cortés et al., 2022). Other clinical trials have shown some variations in survival time and tumor remission response in DS-8201 for HER2 low/positive advanced breast cancer (Modi et al., 2020a; Modi et al., 2020b; Modi et al., 2022). DS-8201 may have potential safety risks according to published data from several studies, as drug-related interstitial lung disease was reported in 10.5% of patients receiving DS-8201 (Kumagai et al., 2020) and 2.2% of the overall population developed lethal (grade 5) interstitial pneumonia (Modi et al., 2022), which is probably due to DS-8201’s high DAR, causing both a decreased *in vivo* circulation half-life and higher toxicities (Li et al., 2019). In addition, almost all related clinical trials have small sample sizes, most of which are small-sample nonrandomized controlled studies with just two relevant randomized controlled studies, leading to a lack of comprehensive and eligible data to evaluate the efficacy and safety of DS-8201. Thus, the purpose of this meta-analysis was to combine all relevant single-arm studies to investigate the efficacy and safety of DS-8201 in the treatment of HER2-low/positive breast cancer, thus providing more therapeutic information about DS-8201 and more options for clinical treatment. We present the following article in accordance with the PRISMA reporting checklist.

## 2 Methods

### 2.1 Search strategy

Seven databases including Embase, PubMed, Cochrane Library, Web of Science, CNKI, VIP database and WanFang data were searched for literature on DS-8201 in the treatment of advanced breast cancer, with the last search completed on 3 November 2022. Additionally, the meeting abstracts of the American Society of Clinical Oncology (ASCO) and the European Society of Medical Oncology (ESMO) were reviewed. The subject terms and free words searched were “Breast Neoplasms” OR “Breast Tumor” OR “Breast Cancer” OR “Breast Malignant Tumor” AND “Ttrastuzumab Deruxtecan” OR “DS-8201a” OR “DS-8201.” Moreover, we manually searched the references of the original literature to avoid missing any relevant articles. The study protocol is available on the Centre for Reviews and Dissemination (CRD) website (registration number CRD42023390316).

### 2.2 Selection criteria

Studies meeting the following inclusion criteria were included in the meta-analysis: 1) The study population included patients with advanced breast cancer diagnosed with HER2 expression based on the ASCO/College of American Pathologists (CAP) guideline criteria for breast cancer (Wolff et al., 2007; Wolff et al., 2013) or HER2 amplification confirmed by FoundationOne CDx; 2) interventions included the treatment of trastuzumab deruxtecan alone; 3) the study type was single-arm research; 4) outcomes included patient-related data such as DCR, ORR, overall survival (OS), progression-free survival (PFS), duration of response (DOR), and adverse events (AEs). Response Evaluation Criteria in Solid Tumors (RECIST) version 1.1 was adopted to evaluate tumor remission (Wolff et al., 2007). Meta-analyses, reviews, conference abstracts, case reports, letters of reply, and animal studies were excluded from the meta-analysis. Two investigators (ZL and SG) independently screened eligible articles according to the inclusion and exclusion criteria, and discrepancies arising during the screening process were settled through discussion between these two researchers (ZL and SG). Any dispute was discussed and resolved by the other four investigators (HX, LL, YG, and SD).

### 2.3 Data extraction and quality assessment

Two investigators (ZL and HX) extracted the data of all included studies independently and subsequently assessed the quality of the included. The extracted data included author name, year of publication, country, sample size, HER2 expression, hormone receptor status, median age, intervention, prior therapy, lines of previous therapy, active brain metastasis or not, median follow-up time, and endpoints reported. Clinical efficacy and safety outcome measures included ORR, DCR, PFS, OS, DOR, the occurrence of any AEs, and ≥ grade 3 AEs. The quality of included studies was evaluated using the methodological index for nonrandomized studies (MINORS) (Slim et al., 2003).

### 2.4 Statistical analysis

STATA 16.0 (StataCorp LP, College Station, TX, United States) was employed for the data analysis of the meta-analysis. The chi-squared test and the I^2^ statistic were used to measure heterogeneity, with *p* < 0.05 considered a statistically significant difference. A random-effects model was adopted if there was significant heterogeneity (*p* < 0.05 and I^2^>50%). Otherwise, a fixed-effects model was used, and a sensitivity analysis was conducted to analyze the stability and reliability of the combined results. Finally, potential publication bias was assessed using Egger’s test and Begg’s test, with *p* < 0.05 indicating a statistically significant difference.

## 3 Results

### 3.1 Study selection

**FIGURE 1:**
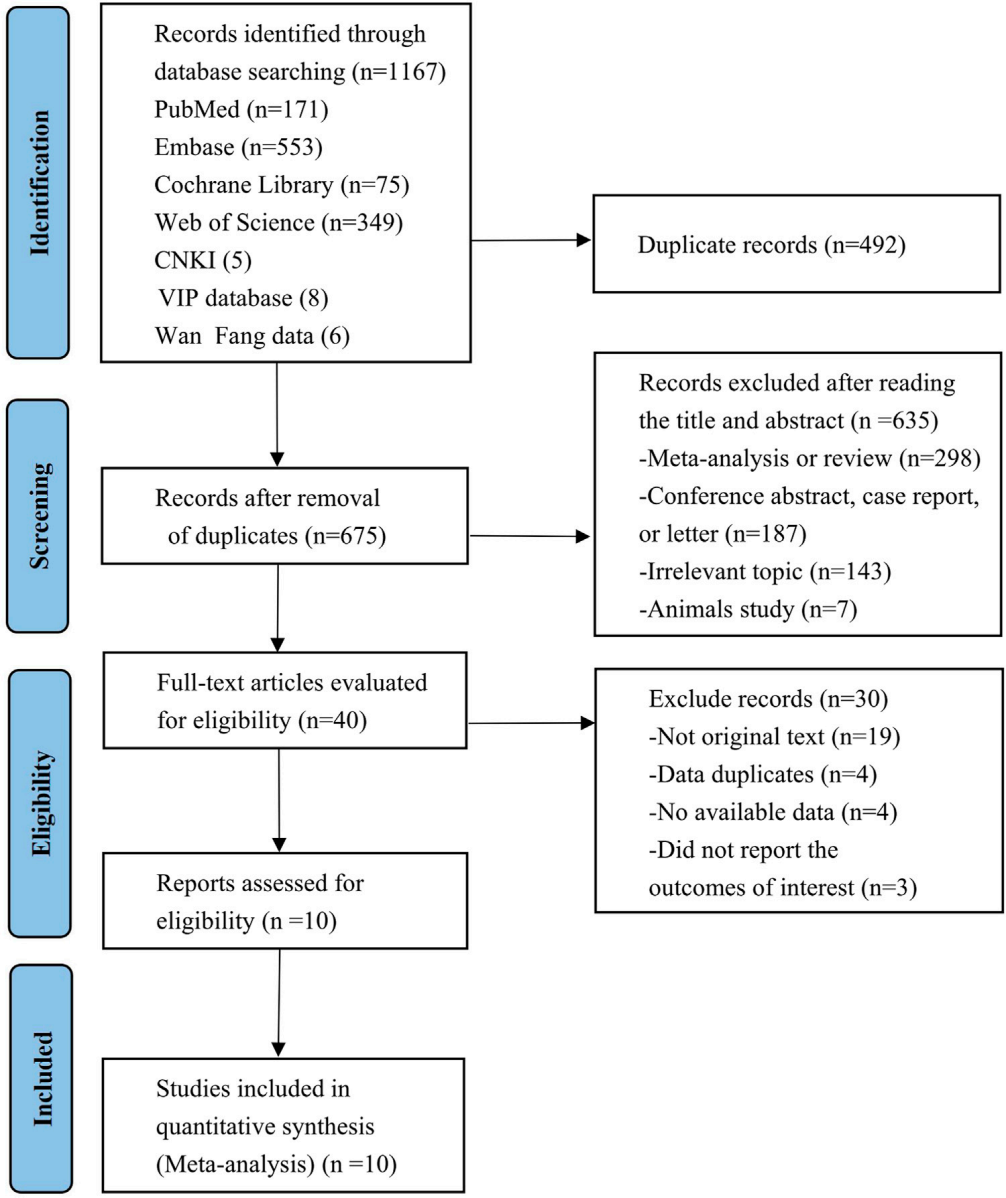
PRISMA flow diagram of the study process. PRISMA, Preferred Reporting Items for Systematic Reviews and Meta-Analysis.

The preliminary search retrieved a total of 1,167 published studies from 7 databases (Embase = 553, PubMed = 171, Web of Science = 349, Cochrane Library = 75, CNKI = 5, VIP database = 8, WanFang data = 6), 40 of which were retained after the elimination of duplicates and the screening of titles and abstracts. After full-text evaluation, 30 studies were further excluded due to a lack of original texts or available data, duplicate data, and failure to report outcomes of concern. Ultimately, a total of 10 studies involving 1,108 patients that met the inclusion criteria were included in this meta-analysis (Chang et al., 2019; Tamura et al., 2019; Modi et al., 2020a; Modi et al., 2020b; Bartsch et al., 2022; Cortés et al., 2022; Modi et al., 2022; Nakajima et al., 2022; Pérez-García et al., 2023; Shimomura et al., 2023). Despite the paper by Chang et al. (2019) not being original, the study was included since the detailed study results were available in the National Clinical Trials Registry. The flowchart of the literature selection process is presented in Figure 1. Among the included patients, 732 were definite HR-positive patients and 308 were explicit HR-negative patients, almost all of whom had experienced two or more types of cancer treatment, with first-line treatment dominated by trastuzumab and/or pertuzumab. Details of each included study are shown in Table 1 and Supplementary Table S1.

**TABLE 1:** Characteristics of the studies included in the meta-analysis.

Study	Year	Nation	Sample size	HER2 expression	Hormone-receptor status		Mean age, years	Intervention	Median follow-up, months	Prior therapy	Endpoints
					Expression	Sample size					
Javier Cortés	2022	Spain	261	positive	Positive	131	54.3 (27.9–83.1)	DS-8201	16.2 (0–32.7)	Trastuzumab; Pertuzumab	ORR, DCR, and AEs
					negative	130				Taxane; Hormone therapy	
										Other anti-HER2 therapy	
Shanu Modi	2020	America	184	positive	positive	97	55.0 (28.0–96.0)	DS-8201	11.1 (0.7–11.9)	Trastuzumab; Pertuzumab	ORR, DCR, DOR, and AEs
					negative	83				TDM-1; Hormone therapy	
					unknown	4				Other anti-HER2 therapy	
Rupert Bartsch	2022	Austria	15	positive	positive	12	69.0 (30.0–76.0)	DS-8201	12.0 (8.0–NR)	Trastuzumab + pertuzumab	ORR, DCR, and AEs
					negative	3				T- DM1; Lapatinib	
Kenji Tamura	2019	Japan	115	positive	positive	81	55.0 (47.0–66.0)	DS-8201	9.9 (6.9–14.3)	Trastuzumab; Pertuzumab	ORR, DCR, and AEs
					negative	33				TDM-1; Lapatinib	
					missing	1				Other anti-HER2 therapy	
Hiromichi Nakajima	2022	Japan	22	positive	positive	15	59.5 (42.0–78.0)	DS-8201	10.1 (8.4–12.0)	Trastuzumab; Pertuzumab	ORR, DCR, and AEs
					negative	7				TDM-1; Lapatinib	
										Other anti-HER2 therapy	
José Manuel Pérez-García	2022	Spain	21	positive	positive	16	53.0 (36.0–77.0)	DS-8201	8.4 (1.4–12.6)	Trastuzumab; Pertuzumab	ORR, DCR, and AEs
					negative	5				TDM-1; Hormone therapy	
										Other anti-HER2 therapy	
Dwan-Ying Chang	2019	China	12	positive	NR	NR	55.0 (36.0–69.0)	DS-8201	NR	NR	ORR, DCR, and AEs
Akihiko Shimomura	2022	Japan	51	positive/low	NR	NR	56.0 (31.0–79.0)	DS-8201	NR	Trastuzumab; Pertuzumab	ORR, DCR, PFS, DOR, and AEs
										TDM-1; Hormone therapy	
										CDK4/6 inhibitors	
										Anthracyclines	
Shanu Modi	2022	America	373	low	positive	333	57.5 (31.5–80.2)	DS-8201	18.4 (17.7–18.9)	CDK4/6 inhibitor	ORR, DCR, PFS, OS, and AEs
					negative	40				Immunotherapy	
										Endocrine therapy	
										Chemotherapy	
Shanu Modi	2020	America	54	low	positive	47	56.6 (33.0–75.0)	DS-8201	NR	CDK4/6 inhibitor; TDM-1	ORR, DCR, OS, and AEs
					negative	7				HER2-targeted therapy	
										Trastuzumab; Pertuzumab	

Notes: NR, not reported; ORR, overall response rate; DCR, disease control rate; PFS, progression-free survival; OS, overall survival; DOR, duration of response; AEs, adverse events.

### 3.2 Quality assessment

All studies were evaluated using the Methodological Index for Non-randomized Studies (MINORS) with 12 evaluation indicators, eight of which were for nonrandomized controlled studies, including clear study objectives, consistency of patients included, and expected data collection, among others (Wolff et al., 2013). Table 2 shows the details of the quality assessment. All included studies were within the low-risk area. The article by Chang et al. (2019) scored slightly lower due to unavailable original publication, but it was included because the data were made accessible in the National Clinical Trials Registry.

**TABLE 2:** Quality assessment of the studies included in the meta-analysis.

Methodological index for non-randomized studies (MINORS) included non-randomized studies	Year	Q1	Q2	Q3	Q4	Q5	Q6	Q7	Q8	Total
Modi et al. (2022)	2022	2	2	2	2	2	2	2	1	15
Modi et al. (2020a)	2020	2	2	2	2	2	1	2	1	14
Modi et al. (2020b)	2020	2	2	2	2	2	2	1	1	14
Cortés et al. (2022)	2022	2	2	2	2	1	2	2	2	15
Bartsch et al. (2022)	2022	2	2	2	2	1	1	2	1	13
Shimomura et al. (2023)	2022	2	2	2	2	2	2	1	1	14
Tamura et al. (2019)	2019	2	2	2	1	2	2	1	1	13
Nakajima et al. (2022)	2022	2	1	2	2	1	1	2	2	13
Pérez-García et al. (2023)	2022	2	2	2	2	1	1	1	2	13
Chang et al. (2019)	2019	2	1	1	1	2	1	1	0	9

Notes: Q1, a stated aim of the study; Q2, inclusion of consecutive patients; Q3, prospective collection of data; Q4, end point appropriate to the study aim; Q5, unbiased evaluation of end points; Q6, follow-up period appropriate to the major end point; Q7, loss to follow-up not exceeding 5%; Q8, prospective calculation of the sample size.

### 3.3 Tumor response

**FIGURE 2:**
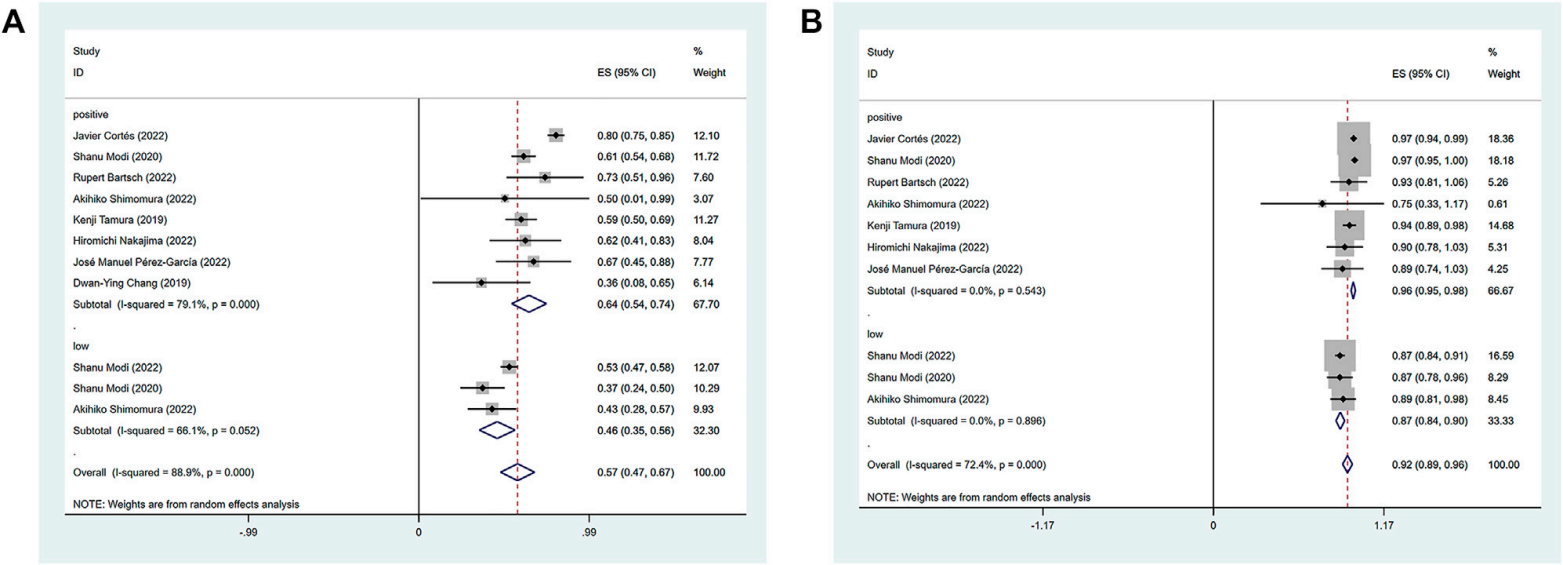
Forest plot for the pooled results of ORR **(A)**, DCR **(B)** in the total *HER2* gene expression subgroup. ORR, objective response rate; DCR, disease control rate; HER2, human epidermal growth factor receptor 2.

**FIGURE 3:**
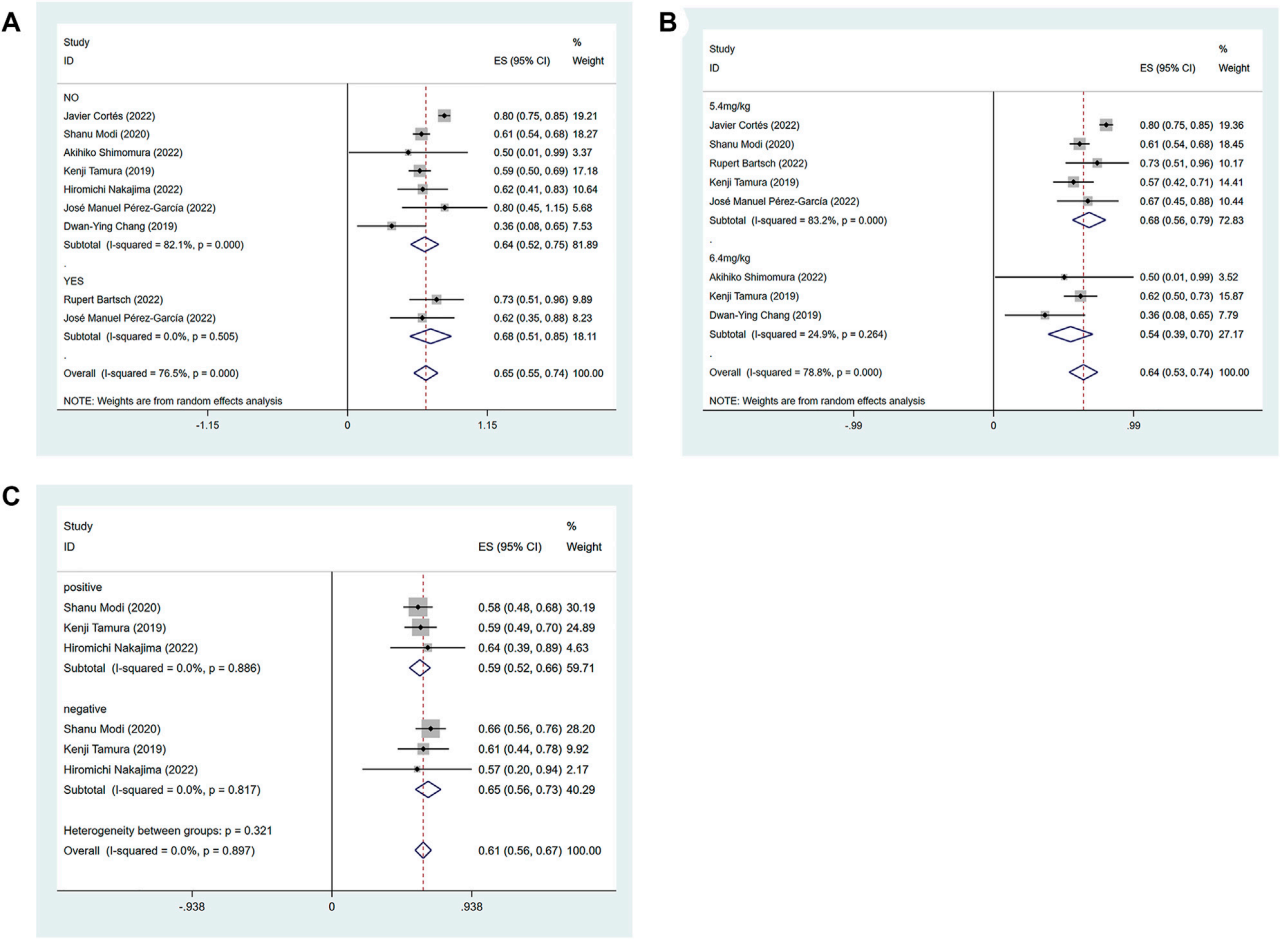
Forest plots of the pooled results of the different subgroup analysis in the HER2-positive expression group. **(A)** ORR for subgroup analysis of whether active brain metastasis, **(B)** ORR for drug dose subgroup analysis, **(C)** ORR for subgroup analysis of hormone receptor status.

**FIGURE 4:**
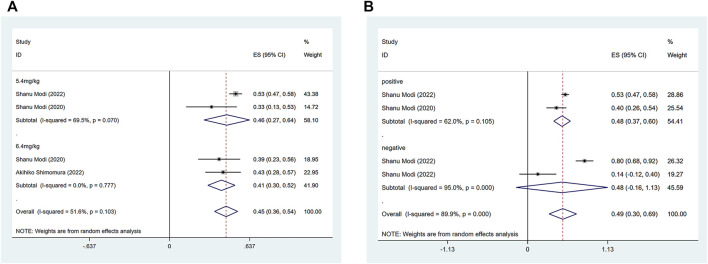
Forest plots of the pooled results of the different subgroup analysis in the HER2 low expression group. **(A)** ORR for drug dose subgroup analysis, **(B)** ORR for subgroup analysis of hormone receptor status.

The efficacy response of DS-8201 for the treatment of breast cancer was demonstrated in all 10 studies included in the analysis, and the ORR ranged from 36% to 80% among the studies. A random-effects model was used due to significant heterogeneity (I^2^ = 88.9%; *p* < 0.001). The analysis showed a pooled ORR of 57% (95% CI: 47%–67%). Subgroup analysis was conducted according to HER2 gene expression. Subgroup analysis showed a pooled ORR of 64% (95% CI: 54%–74%) in HER2-positive patients and 46% (95% CI: 35%–56%) in patients with low HER2 expression (Figure 2A). Because of the differences in clinicopathological features, treatment options and prognosis in HER2 positive and low expressing breast cancers, we analyzed the HER2 positive and HER2 low expressing populations separately again. For the eight studies of HER2-positive breast cancer patients, subgroup analysis was performed according to the presence or absence of active brain metastases, drug dose and hormone receptor status. The subgroup analysis for the presence of active brain metastases showed a pooled ORR of 68% (95% CI: 51%–85%) for patients with active brain metastasis and 64% (95% CI: 52%–75%) for patients without active brain metastasis (Figure 3A). Subgroup analysis by drug doses revealed a pooled ORR of 68% (95% CI: 56%–79%) for patients administered 5.4 mg/kg and 54% (95% CI: 39%–70%) for patients administered 6.4 mg/kg (Figure 3B). The subgroup analysis by hormone receptor status demonstrated a pooled ORR of 59% (95% CI: 52%–66%) for HR-positive patients and 65% (95% CI: 56%–73%) for HR-negative patients (Figure 3C). Meanwhile, although there were only three studies on HER2 low expression breast cancer, we conducted a subgroup analysis by drug dose and hormone receptor status, in the expectation of providing more detailed data for HER low expression breast cancer. Subgroup analysis by drug dose demonstrated a pooled ORR of 46% (95% CI: 27%–64%) for patients with a dose of 5.4 mg/kg and 41% (95% CI: 30%–52%) for patients with a dose of 6.4 mg/kg (Figure 4A); a pooled ORR of 48% in both HR-positive and HR-negative patients was yielded through subgroup analysis by hormone receptor status (Figure 4B). Analysis of DCR data from 10 studies revealed a pooled DCR of 92% (95% CI: 89%–96%) with significant heterogeneity (I^2^ = 72.4%; *p* < 0.001). Subgroup analysis showed a pooled DCR of 96% (95% CI: 95%–98%) in HER2-positive patients and 87% (95% CI: 84%–90%) in patients with low HER2 expression (Figure 2B). Of the 10 studies included in the analysis, only two on HER2 low expression reported a median DOR with significant heterogeneity (I^2^ = 85.5%; *p* = 0.009), but these two papers were ultimately included due to the prominent results. Analysis of their data yielded a pooled median DOR of 11.64 months (95% CI: 4.63–18.64), as shown in Supplementary Figure S1.

### 3.4 Survival

Of the 10 included articles, studies on HER2-positive patients were not used due to incomplete data, insufficient follow-up time and disease progression or death before median survival in some patients. Complete median PFS and OS data could only be extracted from the HER2 low expression studies, with one completely reporting median OS and PFS after DS-8201 administration for all patients and two reporting median OS and PFS after administration for all patients. Regarding median PFS in the low expression group, the pooled median PFS was 9.24 months in a random-effects model (95% CI: 7.54–10.94) (I^2^ = 52.2%; *p* = 0.148), and the combined median OS was 23.87 months (95% CI: 21.56–26.17) using the fixed effects model (I^2^ = 46.6%; *p* = 0.171). The three included studies had a relatively long follow-up period for patients and the overall qualitative assessment of the literature was high and informative. The results of the analysis are detailed in Supplementary Figure S1.

### 3.5 Toxicity

We analyzed the most common and the particularly concerned AEs (all grades and grade ≥ III) in the treatment of HER2-expressing advanced breast cancer with DS-8201 (Table 3; Supplementary Figures S2, S3). Almost all patients experienced grade 1 and 2 adverse reactions, and the three most common adverse reactions reported were nausea, fatigue, and hair loss, with incidences of 62% (95% CI: 48%–76%), 44% (95% CI: 37%–51%), and 38% (95% CI: 32%–44%), respectively. Common hematologic and lymphatic disorders included neutropenia (35%; 95% CI: 27%–43%), anemia (35%; 95% CI: 29%–41%), leukopenia (31%; 95% CI: 23%–39%), thrombocytopenia (25%; 95% CI: 20%–30%). Moreover, the incidence of vomiting (37%; 95% CI: 30%–44%), decreased appetite (36%; 95% CI: 28%–44%), diarrhea (29%; 95% CI: 24%–34%), and constipation (29%; 95% CI: 23%–35%) were relatively high. The incidence of AEs ≥ grade III was significantly reduced, with most of these events occurring at less than 10%. The incidences of nausea, fatigue, and hair loss, the three most common adverse events, were only 5% (95% CI: 3%–6%), 6% (95% CI: 4%–7%), and 0.5% (95% CI: 0%–1%), respectively. Among 1,108 patients with advanced breast cancer, the incidence of drug-related interstitial lung disease or pneumonitis was 13% (95% CI: 11%–15%), with a 1% incidence of AE ≥ grade III.

**TABLE 3:** Adverse events of the studies included in the meta-analysis.

AE	Number of study	All grade			Number of study	≥Grade III		
		ES,% (95CI)	I^2^%	*p*		ES,% (95CI)	I^2^%	*p*
Interstitial lung disease/pneumonitis	9	13 (11–15)	15.7	0.302	8	1 (1–2)	19.3	0.277
Thrombocytopenia	9	25 (20–30)	65.0	0.004	6	6 (4–7)	0.0	0.612
Alopecia	9	38 (32–44)	69.3	0.001	2	0 (0–1)	0.0	0.817
Anemia	10	35 (29–41)	69.1	0.001	8	10 (7–12)	46.6	0.069
Constipation	9	29 (23–35)	68.9	0.001	2	1 (0–2)	0.0	0.750
Decreased appetite	7	36 (28–44)	85.0	0.000	5	2 (1–3)	0.0	0.561
Diarrhea	9	29 (24–34)	63.4	0.005	7	1 (0–2)	32.4	0.180
Fatigue	9	44 (37–51)	77.2	0.000	8	6 (4–7)	0.0	0.784
Leukopenia	7	31 (23–39)	86.0	0.000	6	9 (6–13)	74.1	0.002
Nausea	10	62 (48–76)	96.4	0.000	7	5 (3–6)	6.2	0.380
Neutropenia	10	35 (27–43)	83.5	0.000	8	21 (15–27)	77.7	0.000
Vomiting	9	37 (30–44)	79.8	0.000	4	2 (1–4)	43.7	0.149

Notes: AE, adverse event; ES, effect size.

### 3.6 Sensitivity analysis

A sensitivity analysis was performed on data with I^2^ > 50% to examine the stability of the combined results. According to the sensitivity analysis of all included studies, all combined results with 95% CI in the HER2 positive expression group and HER2 low expression group were not significantly affected by any individual study, which indicates that the overall meta-analysis results are relatively reliable. The results of the sensitivity analysis are detailed in Supplementary Figure S4.

### 3.7 Publication bias

Egger’s and Begg’s tests were used to assess publication bias in this study. In the included studies, the combined ORR (Egger test: 0.342; Begg test: 0.592) and DCR (Egger test: 0.148; Begg test: 0.602) demonstrated no publication bias. The median DOR, PFS, and OS could not be evaluated for publication bias owing to the small number of included studies. No publication bias was observed for the 11 common AEs, but publication bias was present for anemia, diarrhea, leukopenia, and vomiting among the grade ≥ III AEs.

## 4 Discussion

HER2 is a tyrosine kinase receptor membrane glycoprotein encoded by the oncogene ErbB2, whose abnormal expression predisposes normal cells and tissues to carcinogenesis, leading to the proliferation and survival of tumor cells (Arkhipov et al., 2013). HER2 proto-oncogene amplification or protein overexpression has been found in a variety of human tumors, including breast, gastric, ovarian and lung cancers (Iqbal and Iqbal, 2014). Currently, HER2 inhibitors are primarily applied for targeted therapy of HER2-positive breast cancer and are divided into three main categories: monoclonal antibodies (trastuzumab, pertuzumab), tyrosine kinase inhibitors TKI (lapatinib, pyrotinib) and antibody-drug conjugate ADC (TDM-1). Anti-HER2 therapy can inhibit the proliferation of cancer cells and promote apoptosis by inhibiting HER2 signaling, suppressing tumor angiogenesis, and preventing the expression of DNA damage repair genes (Xuhong et al., 2019). Trastuzumab, a conventional treatment for early HER2-positive breast cancer, was shown to reduce the risk of disease recurrence by 40% and the risk of death by 34% in combination with chemotherapy (Slamon et al., 2011). Trastuzumab and docetaxel in combination with pertuzumab prolonged PFS by 6.1 months (*p* < 0.001) and OS by 15.7 months (*p* = 0.0002) compared to the non-combination arm with dual-targeted combination chemotherapy (Baselga et al., 2012a). Lapatinib in combination with trastuzumab monoclonal antibody in neoadjuvant therapy boosted the pathological complete response rate (pCR) rate (Baselga et al., 2012b). All these above have demonstrated the powerful efficacy of anti-HER2 therapy in HER2-positive breast cancer, and trastuzumab + pertuzumab has become the international standard first-line treatment as shown by the NeoSphere clinical study (Gianni et al., 2012; Giordano et al., 2022). Nevertheless, the treatment for locally advanced or metastatic breast cancer is not effective, and most patients will experience disease progression (Harbeck et al., 2019). Compared with previous targeted drugs, antibody-drug conjugates (ADCs), novel antitumor-targeted drugs formed by coupling monoclonal antibodies with targeted effects to chemical drugs with cytotoxic effects (Xu, 2015), have better pharmacokinetic characteristics and cytotoxic effects, typically represented by TDM-1. TDM-1, a second-line agent that has fewer toxic side effects compared to other first-line treatment options (Hurvitz et al., 2018). Phase III clinical results from the EMILI study showed that TDM-1 monotherapy was superior to the combination of capecitabine and lapatinib in terms of median PFS, median OS and ORR. Despite the high efficacy of TDM-1 shown in several clinical trial studies, most patients eventually experience disease progression, and some even have no or mild response after the administration, which may be related to the phenomenon of primary and acquired drug resistance to TDM-1 (Komlodi-Pasztor et al., 2011). For current HER2 low expression breast cancers, several studies have revealed poor prognosis in patients with early or advanced breast cancer (Gilcrease et al., 2009; Rossi et al., 2012; Eggemann et al., 2015) and insignificant treatment effect of traditional anti–HER2 drugs (Boussen et al., 2010; Li et al., 2021). The NSBP B-47, EGF30001, and EGF100151 clinical trials showed that trastuzumab combined with chemotherapy and capecitabine ± lapatinib had little effect on the treatment of breast cancer with low HER2 expression (Press et al., 2008; Zhang et al., 2020). Hence, there is an urgent need for a new drug with good clinical efficacy in both HER2-positive and low expression breast cancers.

In this meta-analysis, we included nine clinical studies and one retrospective analysis encompassing 1,108 patients, and investigated in detail the differences in efficacy based on different subgroups as well as the safety of DS-8201 in the treatment of HER2-low/positive advanced breast cancer. Eight of these studies included patients with HER2-positive advanced breast cancer, three of which involved patients with low HER2 expression. Given the limited clinical data and studies available for HER2 low expression advanced breast cancer, the relatively small number of HER2 low expression studies may still provide additional therapeutic information. The pooled analysis showed that DS-8201 had a promising ORR and DCR, while PFS, OS, and DOR also had certain reference value, showing good efficacy and reliable safety. Despite the HER2 expression, HR status and treatment of the cancer, the pooled results of all studies included an ORR and DCR of 57% (95% CI: 47%–67%) and 92% (95% CI: 89%–96%), respectively. Only the low expression group achieved a median survival time, with pooled median PFS and median OS of 9.24 (95% CI: 7.54–10.94) and 23.87 (95% CI: 21.56–26.17) months, respectively, and the median DOR for patients with low HER2 expression reached 11.64 months. Subgroup analysis of HER2 expression suggested that HER2-positive patients may have a higher ORR (64% vs. 46%) and higher DCR (96% vs. 87%) than patients with low HER2 expression, indicating that DS8201 was likely to have a better effect on HER2-positive advanced breast cancer. Considering the strong effect of anti-HER2 therapy itself on HER2-positive breast cancer and the lack of potent targets for HER2 low expressing breast cancer, the effect of anti-HER2 therapy in HER2 low expressing breast cancer would be weaker. The ORR of nearly 50% and the DCR of nearly 90% in HER2 low expression breast cancer demonstrated the strong and miraculous efficacy of DS8201 as well. Among the eight HER2-positive studies, two involved patients with active brain metastasis, and considering the continued powerful efficacy of DS8201 in these two studies, we conducted a subgroup analysis based on the presence or absence of active brain metastasis. Subgroup analysis indicated that the ORR for the presence of active brain metastasis was 4% higher than that for the absence of active brain metastasis (68% vs. 64%). Previously, local treatments such as whole brain radiotherapy (WBRT), stereotactic radiotherapy (SRT), stereotactic radiosurgery (SRS) and neurosurgery have been the mainstay of treatment for brain metastases, but the prognosis for patients remains generally poor, and neurocognitive decline associated with local treatment also occurs (Soffietti et al., 2017). Based on the strong intracranial and extracranial remission rates of DS-8201, systemic treatment with DS-8201 could be considered to prevent WBRT-related neurocognitive decline. However, the relatively small proportion of patients with active brain metastasis in our study may have overstated the efficacy of DS8201, and more studies with larger samples are needed. The current recommended dose of DS-8201 is 5.4 mg/kg or 6.4 mg/kg in a 3-week cycle (Doi et al., 2017), and we wondered whether different doses of DS-8201 would produce varied efficacy. Subgroup analysis revealed that in the HER2-positive group, the ORR was higher in the 5.4 mg/kg group than in the 6.4 mg/kg group (68% vs. 54%); in the HER2 low expression group, the ORR was higher in the 5.4 mg/kg group than in the 6.4 mg/kg group (46% vs. 41%), both demonstrating a superior treatment effect of the dose of 5.4 mg/kg. Therefore, it is advisable to recommend a dose of 5.4 mg/kg of DS-8201 to patients. It was interesting to note that subgroup analysis by hormone receptor status revealed that in the HER2-positive group, HR-negative patients had a higher ORR than HR-positive patients (65% vs. 59%), demonstrating that DS-8201 may have better efficacy for HR-/HER + patients; in the HER2 low expression group, the ORR was 48% for both HR+ and HR-patients, but the HER2 low expression group was included in few studies, and further studies are required for validation in the future. Overall, in terms of clinical efficacy, DS-8201 is a new generation of ADC that offers new hopes for patients with HER2 low expression advanced breast cancer, and HER2-positive data also support the better clinical efficacy of DS-8201 compared to conventional HER2-positive regimens. Our analysis of AEs showed that almost all patients experienced at least 1 reaction, with the three most common adverse reactions being nausea (62%, 95% CI: 48%–76%), fatigue (44%, 95% CI: 37%–51%), and hair loss (38%, 95% CI: 32%–44%). The incidence of AES ≥ grade III was reduced significantly, and the incidence of the majority of these events was below 10%. The incidence of the three most common AEs, nausea, fatigue and hair loss, was only 5% (95% CI: 3%–6%), 6% (95% CI: 4%–7%), and 0.5% (95% CI: 0%–1%), respectively. For the important adverse event of drug-related interstitial lung disease or pneumonitis, the incidence of disease was 13% (95% CI: 11%–15%), with only 1% incidence of AE ≥ grade III. Based on these results, we surmised that DS-8201 has an acceptable safety profile for patients with HER2-low/positive advanced breast cancer.

DS-8201 and TDM-1 belong to the same class of ADCs. The phase III clinical studies showed that ORR more than doubled in the DS-8201 group compared to the TDM-1 group (79.7% vs. 34.2%) for patients with HER2-positive breast cancer; moreover, the median PFS for patients in the DS-8201 treatment group presented a more than 3-fold improvement compared to the TDM-1 control group (Cortés et al., 2022). This randomized controlled study demonstrated that DS-8201 is superior to TDM-1 and may replace TDM-1 as the standard second-line treatment option for HER2-positive advanced breast cancer. In another study of patients with breast cancer and low HER2 expression, the ORR of the DS-8201 group was 52.6% compared with that of the physician-recommended chemotherapy group, and that of the control group was only 16.3%. The median PFS of DS-8201 was 5 months higher than the physician-recommended chemotherapy group, and DS-8201 had a 14.8% lower incidence (52.6% vs. 67.4%) of grade 3 or higher adverse reactions than the control group (Modi et al., 2022). In February 2015, the FDA approved CDK4/6 inhibitors for the treatment of patients with metastatic hormone receptor (HR)-positive, HER2-negative breast cancer. In China, a targeted breakthrough therapy for the treatment of HR+/HER2-advanced breast cancer has been lacking for decades, and the approval of Palbociclib in combination with aromatase inhibitor (AI) as a first-line treatment for women with HR+/HER2 advanced breast cancer in 2018 has become a milestone. Cyclin-dependent kinases 4 and 6 (CDK4/6) inhibitors, including palbociclib, ribociclib and abemaciclib, block DNA synthesis and cell proliferation by selectively inhibiting CDK4/6, interfering with the cell cycle protein D-CDK 4/6 retinoblastoma pathway, while avoiding the pan-CDK inhibitors associated with severe cytotoxicity, restoring cell cycle control and blocking tumor cell proliferation (Gao et al., 2020). Nowadays, the combination of CDK 4/6 inhibitors and endocrine therapy has become the standard of treatment for patients with HR+/HER2-advanced breast cancer (Gradishar et al., 2020). A multicenter retrospective analysis based on three hospitals in China confirmed the clinical benefit of palbociclib in combination with endocrine therapy in Chinese patients, regardless of metastatic sites (Zhang et al., 2022). CDK4/6 inhibitors have been demonstrated to improve the prognosis of HR+/HER2-breast cancer; however, not all patients respond to CDK4/6 inhibitors, and even patients sensitive to CDK4/6 inhibitors may develop resistance. The development of intrinsic or acquired resistance may limit the efficacy of these treatments (Pandey et al., 2019). Therefore, exploring the sensitivity or resistance mechanisms of CDK4/6 inhibitors becomes the next target in HR+/HER2-breast cancer. In a study with a sample size of 373, for patients with HR+/HER2 low expression advanced breast cancer at the end of DS-8201 cyclic treatment, median PFS was found to be longer in patients who had not previously received CDK4/6 inhibitors than in those who had been previously administrated (11.7 months vs. 10.0 months) (Modi et al., 2022). A subgroup analysis of another study with a sample size of 54 patients with HR+/HER2 low expression advanced breast cancer after DS-8201 cyclic treatment showed that ORR was 9.6% higher in patients previously treated with CDK4/6 inhibitors than in those who did not receive CDK4/6 inhibitors (43.8% vs. 34.2%) (Modi et al., 2020b). These two contradictory results may be due to small sample sizes or related to the characteristics of the patients, and more subsequent studies with larger sample sizes are needed to validate these results. A pooled analysis of adverse events showed a promising safety profile for DS-8201, but drug-related interstitial lung disease or pneumonitis still requires significant attention. The pooled analysis of nine phase I/II DS-8201 monotherapy treatments, including multiple carcinomas, found drug-related ILD/pneumonia in 15.4% (177/1,150) of patients, with 15 (1.3%) patients suffering grade 3 or 4 events and 25 patients suffering (2.2%) grade 5 events (Powell et al., 2022). Previous preclinical studies of DS-8201 in combination with anti-PD-1 antibodies in tumor-bearing mice expressing human HER2 antigens found that the anti-tumor effects of this combination treatment modality were greater than either monotherapy (Iwata et al., 2018). Therefore, based on several preclinical and clinical observational studies (Saini et al., 2021), ADC combination immunotherapy is currently in full swing, and more relevant studies and data are desired to further investigate the clinical efficacy and safety of DS-8201 in combination with immunosuppressive drugs.

There are some limitations to our current meta-analysis. First, there were few studies on patients with HER2 low expression, which were insufficient to explore the actual efficacy of DS-8201 in the HER2 low expression group and perform subgroup analysis, especially HR+/HER low expression patients, and we could only provide information about the clinical treatment of DS-8201. Second, we included nonrandomized controlled results with small sample sizes and could only assess the clinical efficacy and safety of DS-8201, but no definitive conclusions can be drawn. Third, most of the included studies had a short follow-up period, and survival time could not be accurately assessed. Therefore, large-scale randomized controlled trials should be conducted to confirm the clinical efficacy of DS-8201 in patients with advanced breast cancer with HER2 expression so as to provide more options for clinical treatment.

## 5 Conclusion

In summary, our meta-analysis confirmed the efficacy and safety of DS-8201 in patients with HER2-low/positive advanced breast cancer as a promising clinical treatment option and reminded us to enhance surveillance for drug-related interstitial lung disease or pneumonitis. However, there are limitations in the clinical data, and future large-sample, and multicenter randomized controlled trials are needed to further validate our findings.
