# Supplementary material for: Efficacy and safety of trastuzumab deruxtecan in the treatment of HER2-low/positive advanced breast cancer: a single-arm meta-analysis
Source: Front Pharmacol. 2023 Jun 22;14:1183514. doi: 10.3389/fphar.2023.1183514 (PMC10324614; doi:10.3389/fphar.2023.1183514)
Supplement: Supplementary file 2 [file DataSheet1.docx]

# Supplementary materials

**TableS1** Other characteristics of the studies included in the meta-analysis

| **Study** | **Year** | **Sample size** | **Nation** | **Median lines of previous therapy** | **Previous therapy lines** | | **Active brain metastasis** | |
| --- | --- | --- | --- | --- | --- | --- | --- | --- |
|  |  |  |  |  | **Number** | **Sample size** | **YES/NO** | **Sample size** |
| Javier Cortés | 2022 | 261 | Spain | 1 (0-16) | 0 or 1 | 132 | NO | 261 |
|  |  |  |  |  | 2 | 56 |  |  |
|  |  |  |  |  | ≥3 | 73 |  |  |
| [Shanu Modi](https://pubmed.ilibs.cn/?term=Modi+S&cauthor_id=35665782) | 2020 | 184 | America | NR | NR | | NO | 184 |
| Rupert Bartsch | 2022 | 15 | Austria | 2 (1-5) | NR | | YES | 15 |
| [Kenji Tamura](https://pubmed.ilibs.cn/?term=Tamura+K&cauthor_id=31047803) | 2019 | 115 | Japan | NR | NR | | NO | 115 |
| [Hiromichi Nakajima](https://pubmed.ilibs.cn/?term=Nakajima+H&cauthor_id=34999427) | 2022 | 22 | Japan | NR | NR | | NO | 22 |
| [José Manuel Pérez-García](https://pubmed.ilibs.cn/?term=P%C3%A9rez-Garc%C3%ADa+JM&cauthor_id=35639825) | 2022 | 21 | Spain | NR | 0 or 1 | 3 | YES | 13 |
|  |  |  |  |  | 2 | 5 |  |  |
|  |  |  |  |  | ≥3 | 13 |  |  |
| Dwan-Ying Chang | 2019 | 12 | China | NR | NR | NR | NO | 12 |
| [Akihiko Shimomura](https://pubmed.ilibs.cn/?term=Shimomura+A&cauthor_id=36164935) | 2022 | 51 | Japan | NR | NR | NR | NO | 51 |
| [Shanu Modi](https://pubmed.ilibs.cn/?term=Modi+S&cauthor_id=35665782) | 2022 | 373 | America | 3 (1-9) | 0 or 1 | 39 | NO | 373 |
|  |  |  |  |  | 2 | 100 |  |  |
|  |  |  |  |  | ≥3 | 234 |  |  |
| [Shanu Modi](https://pubmed.ilibs.cn/?term=Modi+S&cauthor_id=35665782) | 2020 | 54 | America | 7.5 (2-16) | NR | | NO | 54 |

Note: NR, not reported


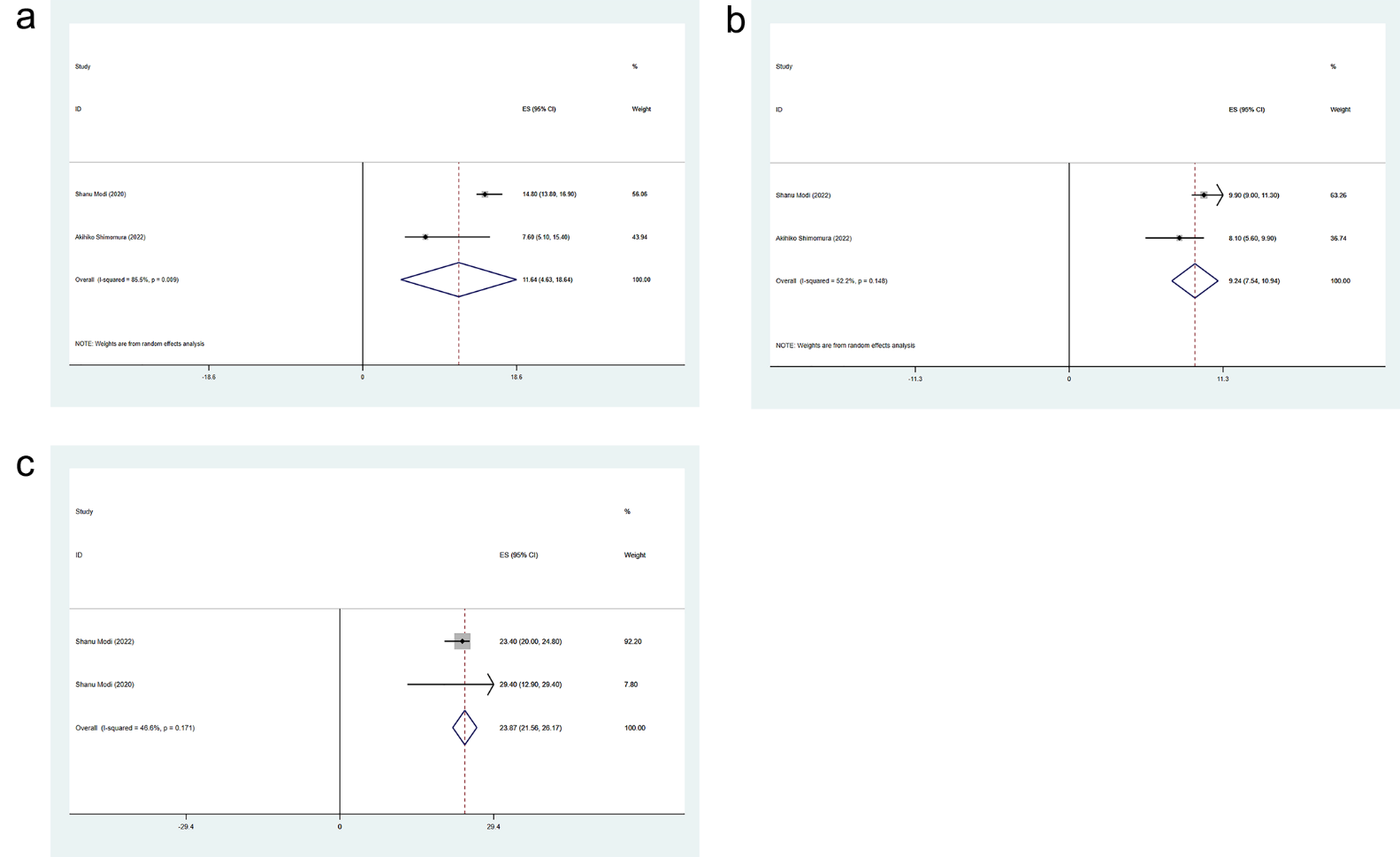


Figure S1 Forest plot about the pooled results of DOR (a), PFS (b) and OS (c) in the HER2 low expression group. DOR, duration of response; PFS, progression-free survival; OS, overall survival.


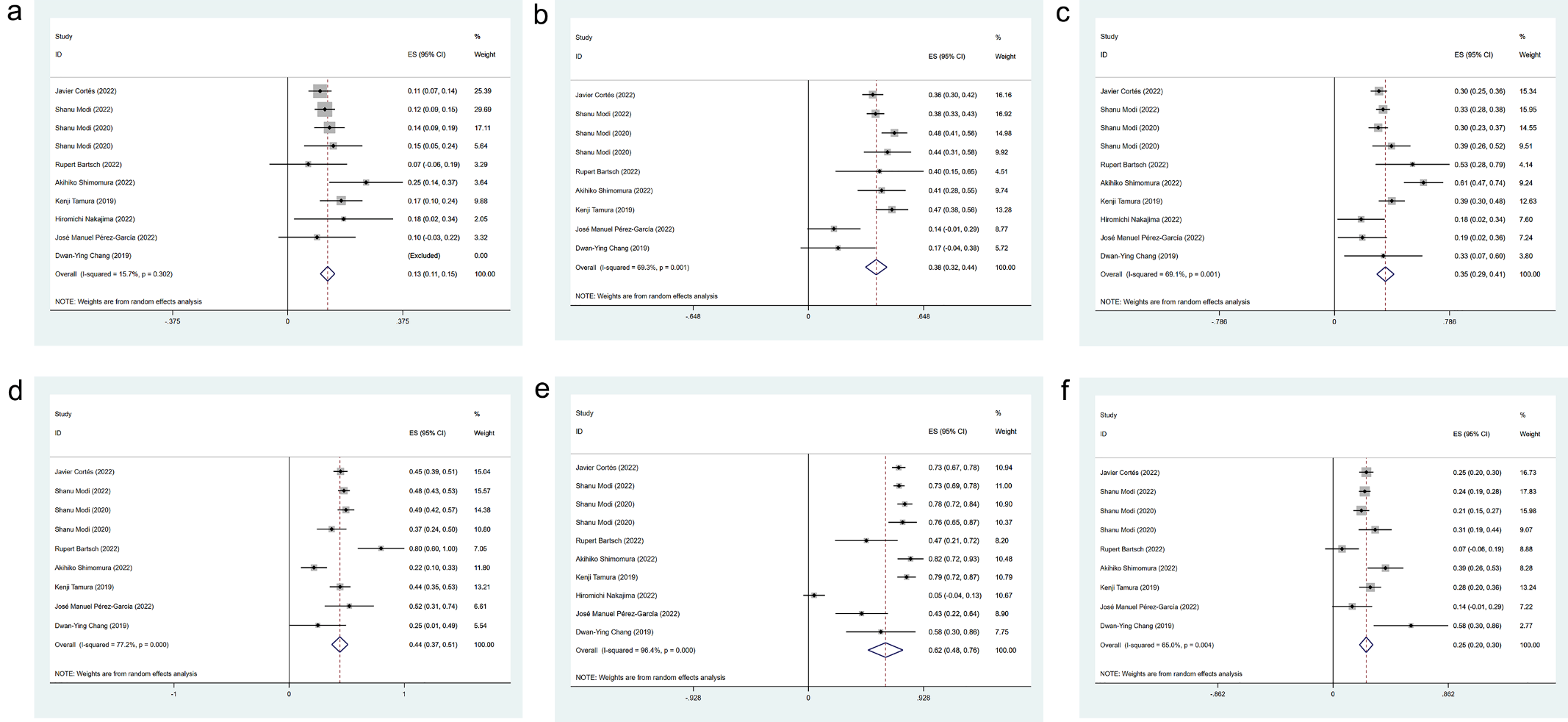


Figure S2 Forest plot about the pooled results of any-grade adverse events. (a)Interstitial lung disease or pneumonitis, (b) Alopecia, (c) Anemia, (d) Fatigue, (e) Nausea, (f) Thrombocytopenia.


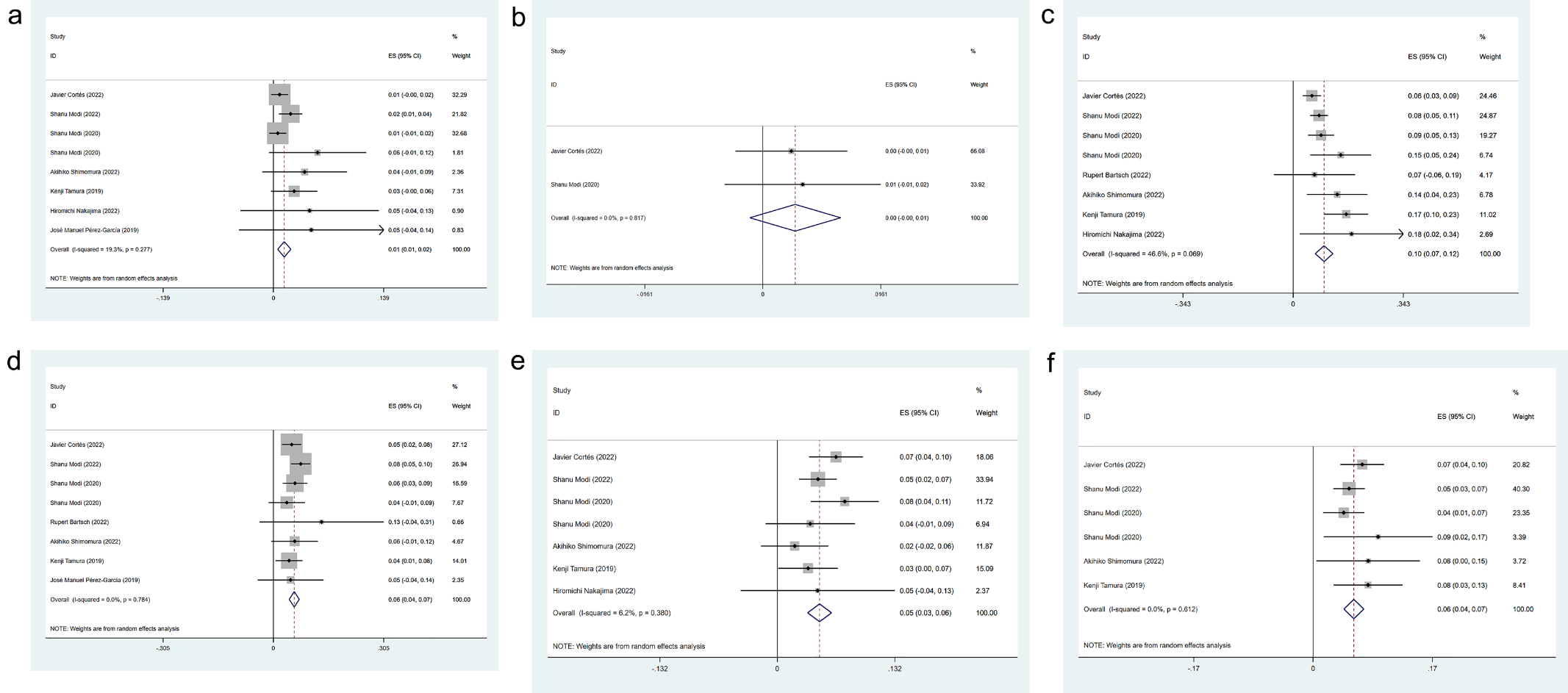


Figure S3 Forest plot about the pooled results of Grade III and higher adverse events. (a)Interstitial lung disease or pneumonitis, (b) Alopecia, (c) Anemia, (d) Fatigue, (e) Nausea, (f) Thrombocytopenia.


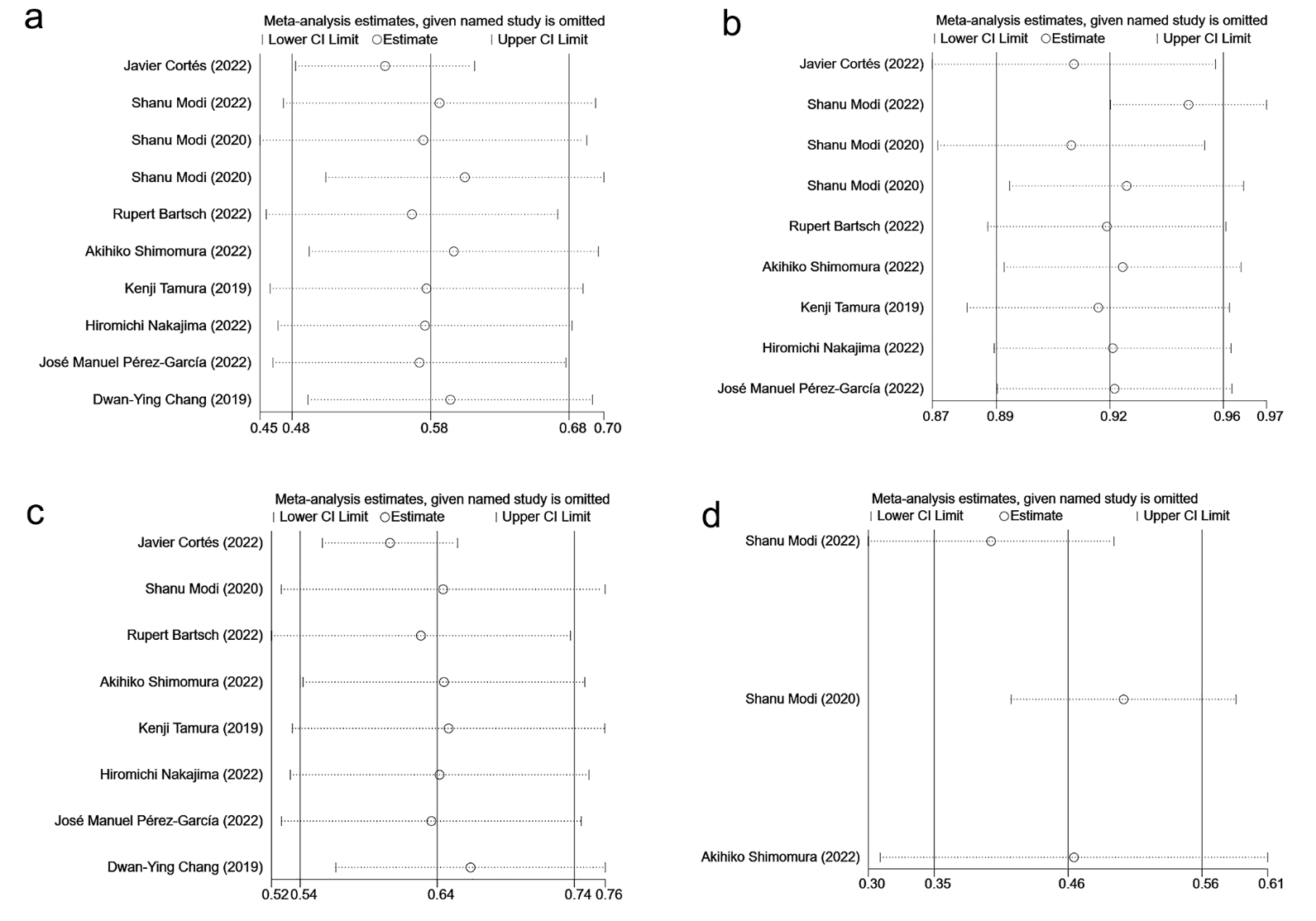


Figure S4 Sensitivity analysis. (a) Sensitivity analysis for ORR in total studies, (b) Sensitivity analysis for DCR in total studies, (c) Sensitivity analysis for ORR in HER2-postive group, (d) Sensitivity analysis for ORR in HER2 low expression group. ORR, objective response rate; DCR, disease control rate.
